# MRI findings of low-grade fibromyxoid sarcoma: a case report and literature review

**DOI:** 10.1186/s12891-018-1976-z

**Published:** 2018-02-26

**Authors:** Yali Yue, Yongkang Liu, Lina Song, Xiao Chen, Yaohui Wang, Zhongqiu Wang

**Affiliations:** 1 Department of Radiology, Affiliated Hospital of Nanjing University of Chinese Medicine, 155 Hanzhong Road, Nanjing, Jiangsu 210029 People’s Republic of China; 2 Department of Pathology, Affiliated Hospital of Nanjing University of Chinese Medicine, Nanjing, Jiangsu 210029 China

**Keywords:** Magnetic resonance imaging, Anterior pelvic wall, Low-grade fibromyxoid sarcoma

## Abstract

**Background:**

Low-grade fibromyxoid sarcoma (LGFMS) is a distinctive slow growing soft tissue neoplasm, mostly affecting young individuals with no gender difference. It usually arises in deep soft tissue of the lower limbs and trunk, but few cases of LGFMS located in pelvis have been reported.

**Case presentation:**

We describe the magnetic resonance imaging(MRI) features of LGFMS located in the anterior pelvic wall of a 21-year-old female and correlate them with clinicopathological features. The tumor was completely resected and there is no recidivism during the follow-up one year.

**Conclusions:**

We report on the radiological findings of LGFMS with histological correlation. Awareness of the imaging features may be useful for the diagnosis of LGFMS and helpful to distinguish among mimics.

## Background

Low-grade fibromyxoid sarcoma (LGFMS) is a slow growing sarcoma that originally described by Evans [1]. LGFMS is defined by the World Health Organization (WHO) as a malignant fibroblastic/myofibroblastic tumor usually arise from deep soft tissue with potential for recurrence and late metastatic spread [2, 3]. The incidence is estimated 0.18 per million, accounting for 0.6% of all soft tissue sarcomas [4]. Due to the relative rarity, it is difficult to distinguish LGFMS from other mesenchymal tumors and make accurate diagnosis for the radiologists. To make a deep understanding of this kind of disease, we describe MRI findings of a 21-year-old woman LGFMS patient and review relevant literature. This study may be conducive to future diagnosis and differential diagnosis of LGFMS for radiologists.

## Case presentation

A 21-year-old female with a palpable painless mass in her lower abdomen which was gradually increasing in size was admitted to our institution. Physical examination demonstrated a firm, non-tender and non-movable mass measuring 8 × 9 cm in size protruding through the anterior pelvic wall. The index of hemotology and biochemistry were all in the normal scope. The patient has no history of injury or surgery, and no family history was identified.

**Fig. 1:**
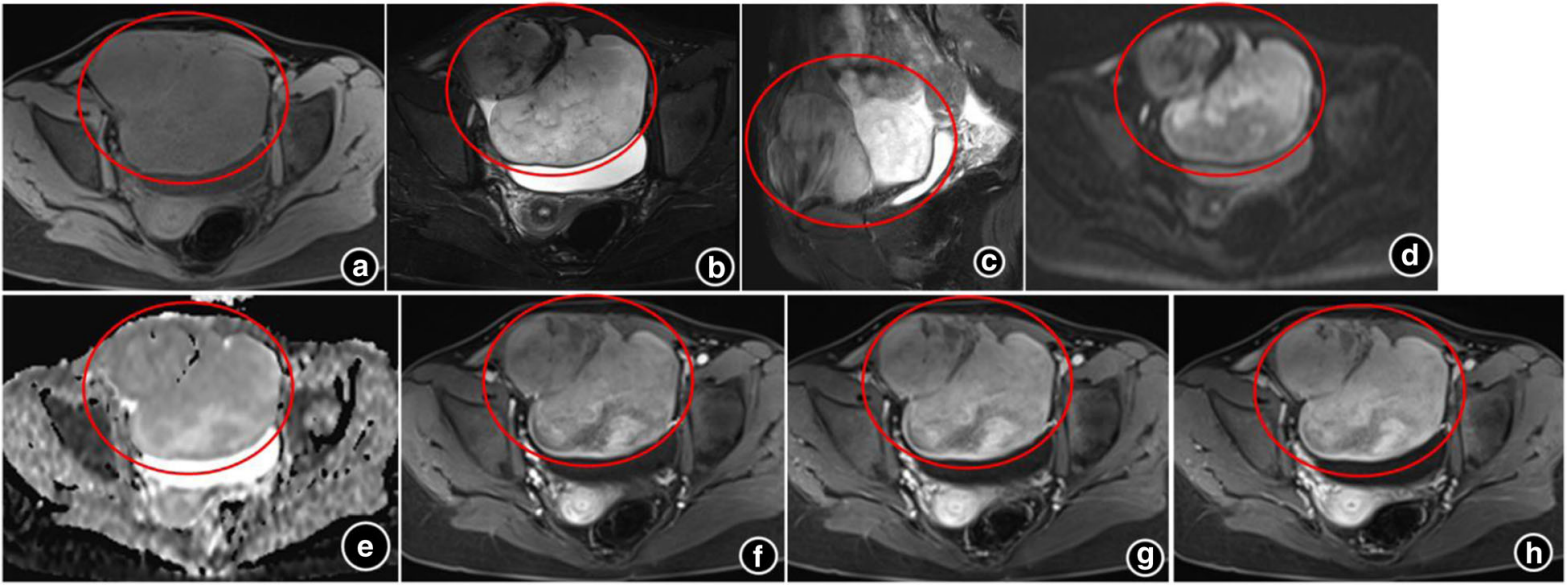
The magnetic resonance imaging findings of low grade fibromyxoid sarcoma. **a**: T1-weighted MR images; **b**: T2-weighted MR images; **c**: Sagittal T2WI showed that the rectus abdominis was open with umbrella shape; **d**: The tumor showed high signal intensity on diffusion weighted imaging; **e**: The tumor showed low signal intensity on apparent diffusion coefficient (ADC) map; **f**-**h**: On Gadolinium-enhanced T1-weighted MR images with fat suppression at arterial (**f**), portal (**g**) and delayed phases (**h**), the tumor showed heterogeneous enhancement

**Fig. 2:**
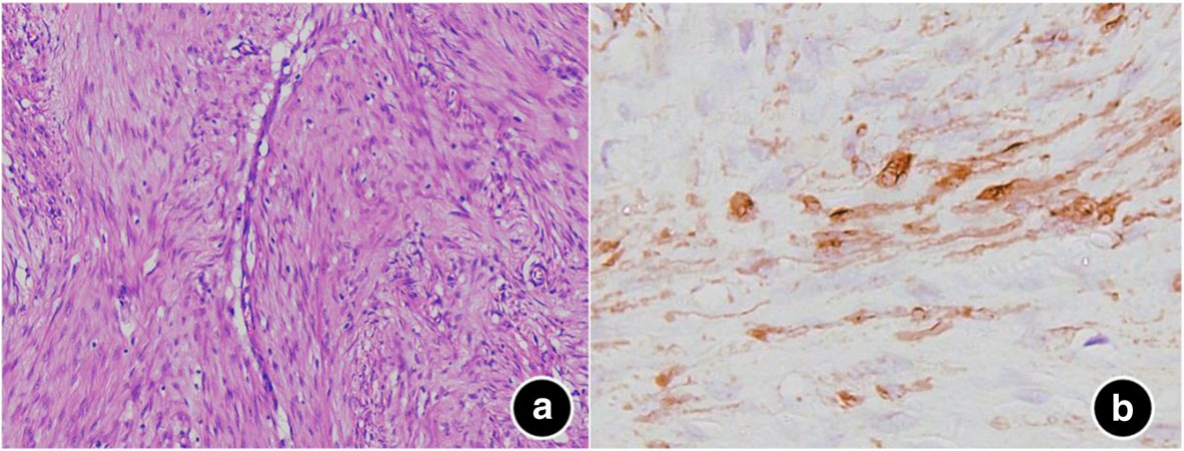
Microscopic findings of low grade fibromyxoid sarcoma. **a**: Hematoxylin-eosin staining (magnification, × 100); **b**: immunochemical staining for smooth muscle actin (SMA) (magnification, × 400)

The nosocomial MRI revealed a well-defined, 12 × 10 × 9 cm-sized, lobulated mass within the mid-pelvis. The mass exhibited isointensity relative to muscle in fat-supressed T1-weighted MR image (Fig. 1a) and heterogeneous relatively hyperintensity with a little stip and patchy hypointensity inside on fat-suppression T2-weighted MR image (Fig. 1b). Meanwhile, circinate low signal intensity can be found around the tumor, indicating that there was a capsule or pseudocapsule. Sagittal T2WI showed that the rectus abdominis was open with umbrella shape (Fig. 1c). The tumor showed restricted diffusion on diffusion weighted imaging (DWI) (Fig. 1d) and apparent diffusion coefficient (ADC) map (Fig. 1e), which presented as heterogeneous hyperintensity and hypointensity, respectively. It also showed heterogeneous enhancement on fat-suppression T1WI after the contrast agent injected (Fig. 1f-1h). The area of higher signal intensity exhibited more obvious enhancement compared with lower signal intensity area on T2WI. At surgery, the mass was located at the symphysis ossium pubis, invading the lower right rectus abdominis and the bottom of the bladder. It was well circumscribed, firm and lobulated appearance. The cut surface of the lesion was pale white and glistening with neither necrosis nor hemorrhage. Microscopic examination identified alternating fibrous and myxoid stroma areas inside the tumor, the tumor cells arranging in a whorled growth pattern with curvilinear capillaries (Fig. 2a). Immunohistochemically, the tumor cells were positive for smooth muscle actin (SMA) (Fig. 2b). MRI findings combined with histopathological analysis made a final diagnosis of LGFMS. The patient was discharged from hospital 10 days after the operation. During one year of follow-up, the patient was in a good status without evidence of recurrence.

## Discussion and conclusions

LGFMS was first depicted by Evans in 1987 [1], who subsequently reported the clinicopathologic features of these tumors in subsequent studies [5]. It has been shown that LGFMS seem to occur mainly in young adults without difference in gender (median age 34 years old) [6]. LGFMS usually occur in deep soft tissue, manifestating as a slow-growing, non-tender and solid tumor [5, 6]. It usually presents as a well circumscribed soft tissue mass, without hemorrhage, nodularity or infiltrative border, and is usually favor of a benign lesion. The microscopic appearance of LGFMS is composed of alternative fibrous and myxoid zones and spindle cells arranging in a swirling pattern with no obvious atypia [7, 8].

Radiological examination plays a crucial part in both diagnosis and treatment of this kind of disease. CT and MRI can visually characterize lesions’ morphological features, textural features, and relationships with adjacent structures. On CT or MRI, LGFMS is solitary and well circumscribed, which is similar to the lesion in our study, although it may present as multiple infiltrating masses at recurrence [9]. Several previous case reports have described CT and MRI observations of LGFMS. On plain CT scan, the fibrous component has been depicted as isodense while the myxoid component has been depicted as hypodense [6]. Conventional and advanced MR techniques can be more helpful than CT in the assessment of various muscle disorders for its further subtle multi-sequence imaging [10]. MRI performances of LGFMS have previously been reported [11, 12]. On MRI, LGFMS is inhomogeneous, owing to its two distinct internal zones: myxoid and fibrous. Generally, the low T1/T2 signal intensity and slight enhancement correspond to fibrous components [13, 14]. The low T1 signal intensity, high T2 signal intensity and variable enhancement after gadolinium injection correspond to the myxoid components [6, 15]. In our case, the tumor had an inhomogeneous appearance on T1WI and T2WI. The internal components were hypointense to muscle on T2WI. The relative higher signal intensity zone showed more intense enhancement compared with lower signal intensity on T2WI, as formerly reported [11]. Additionly, DWI also plays an important part in differentiating malignant from benign lesions and necrotic tumors from infections [16, 17]. DWI is a noninvasive method used to measure the motion of water molecules and can be quantified as ADC. Both soft tissue tumors and infectious diseases may present hyperintense signal on DWI and hypointense signal on the ADC map. However, hyperintensity relative to skeletal muscle is more frequent in malignant than benign tumors on DWI, ADCs of malignant tumors are remarkably lower than benign tumors. Most necrotic tumors show higher ADC values and low-to-intermediate signal intensity on DWI, whereas abscesses has lower ADC values and higher signal intensity on DWI in contrast.

Although MRI and CT can detect the fibrous and myxoid components of the tumor, sometimes, it is very difficult to distinguish theses lesions from LGFMS only depending on imageological examinations. The definite diagnosis depends on histopathological examination. Immunohistochemical staining showed positive expression of vimentin in the tumor cells, but SMA, β-catenin, desmin, S100, cytokeratin, CD34 and CD56 were negative [18]. In the present case, the LGFMS was negative for vimentin and positive for SMA.

Various similar benign or malignant soft tissue lesions should be considered in the differential diagnosis of LGFMS, of which has the significant potential for recurrence and late metastatic spread. Low-grade myxofibrosarcoma is the most important malignant entities among various mimics. It is usually confused with LGFMS, partially due to similar nomenclature. LGFMS commonly locate in the deep soft tissue which tend to occur in young adults, while low-grade myxofibrosarcoma primarily locate in subcutaneous layer of the extremities of elderly individuals. Most myxofibrosarcoma cases histologic expressions include: homogeneous myxoid zone, lack of alternative fibrous zones, pleomorphic cells, and hyperchromatic nuclei [19]. Intra-abdominal fibromatosis may have myxoid areas and pluricellular areas with a fascicular pattern of growth just mimic LGFMS [20]. However, unlike LGFMS, fibromatosis is a poorly-defined tumor with infiltrative borders and exhibits myofibroblastic differentiation morphologically and immunohistochemically [21]. Nodular fasciitis can also have variably myxoid and fibrous zones with well circumscribed boundary [20]. However, histological features including a loose storiform pattern of growth, myxoid degeneration and extravasation of lymphocytes and erythrocytes, which are characteristics of nodular fasciitis, are absent in LGFMS. LGFMS should also be differentiated from Morel-Lavallee Lesions, which is a post-traumatic soft tissue degloving injury. The appropriateclinical history and a fluid collection in a typical location are useful in determining the diagnosis of Morel-Lavallee Lesions [22].

In conclusion, we reviewed previously publications combining with the current 21-year-old female case, and some relative characteristics of LGFMS can be summarized as following. LGFMS is an uncommon soft tissue tumor that usually arise from deep soft tissue of young individuals and has high risk of recurrence and late metastatic spread. Fibrous component areas show low T1/T2 signal intensity. Myxoid component areas show low T1 signal intensity, high T2 signal intensity and variable enhancement. Awareness of the above may be useful for the diagnosis of LGFMS.

## Abbreviations

ADC: Apparent diffusion coefficient
DWI: Diffusion weighted imaging
LGFMS: Low-grade fibromyxoid sarcoma
MRI: Magnetic resonance imaging
SMA: Smooth muscle actin
WHO: World health organization

## References

[1] Evans HL (1987). Low-grade fibromyxoid sarcoma. A report of two metastasizing neoplasms having a deceptively benign appearance. Am J Clin Pathol.

[2] Kurisakiarakawa A, Suehara Y, Arakawa A, Takagi T, Takahashi M, Mitani K, Kaneko K, Yao T, Saito T (2014). Deeply located low-grade fibromyxoid sarcoma with FUS-CREB3L2 gene fusion in a 5-year-old boy with review of literature. Diagn Pathol.

[3] Doyle LA, Möller E, Dal CP, Fletcher CD, Mertens F, Hornick JL (2011). MUC4 is a highly sensitive and specific marker for low-grade fibromyxoid sarcoma. Am J Surg Pathol.

[4] Marettynielsen K, Baerentzen S, Keller J, Dyrop HB, Safwat A (2013). Low-Grade Fibromyxoid Sarcoma: Incidence, Treatment Strategy of Metastases, and Clinical Significance of the FUS Gene. Sarcoma.

[5] Evans HL (1993). Low-grade fibromyxoid sarcoma. A report of 12 cases. American. J Surg Pathol.

[6] Miyake M, Tateishi U, Maeda T, Arai Y, Seki K, Hasegawa T, Sugimura K (2006). CT and MRI features of low-grade fibromyxoid sarcoma in the shoulder of a pediatric patient. Radiat Med.

[7] Evans HL (2011). Low-grade fibromyxoid sarcoma: a clinicopathologic study of 33 cases with long-term follow-up. Am J Surg Pathol.

[8] Kim SY, Kim MY, Hwang YJ, Han YH, Seo JW, Kim YH, Cha SJ, Hur G (2005). Low-grade fibromyxoid sarcoma: CT, sonography, and MR findings in 3 cases. J Thorac Imaging.

[9] Hwang S, Kelliher E, Hameed M (2012). Imaging features of low-grade fibromyxoid sarcoma (Evans tumor). Skelet Radiol.

[10] Kumar Y, Wadhwa V, Phillips L, Pezeshk P, Chhabra A (2016). MR imaging of skeletal muscle signal alterations: systematic approach to evaluation. Eur J Radiol.

[11] Kim SK, Jee WH, Lee AW, Chung YG (2011). Haemorrhagic low-grade fibromyxoid sarcoma: MR findings in two young women. Br J Radiol.

[12] Sargar K, Kao SC, Spunt SL, Hawkins D, Parham DM, Coffin C, Mccarville MB (2015). MR and CT imaging of low grade Fibromyxoid sarcoma in children: a report from Children’s oncology group study ARST0332. AJR Am J Roentgenol.

[13] Arnaoutoglou C, Lykissas MG, Gelalis ID, Batistatou A, Goussia A, Doukas M, Xenakis TA (2010). Low grade fibromyxoid sarcoma: a case report and review of the literature. Journal of Orthopaedic Surgery & Research.

[14] Lee BJ, Park WS, Jin JM, Ha CW, Lee SH (2009). Low grade fibromyxoid sarcoma in thigh. Clinics in Orthopedic Surgery.

[15] Bajpai J, Shukla S, Jah M, Singh AK, Goel M, Mourya A, Sachdeva N (2014). Low-grade fibromyxoid sarcoma around the knee involving the proximal end of the tibia and patella: a rare case report. Oncol Lett.

[16] Kumar Y, Khaleel M, Boothe E, Awdeh H, Wadhwa V, Chhabra A (2016). Role of diffusion weighted imaging in musculoskeletal infections: current perspectives. Eur Radiol.

[17] Lee SY, Jee WH, Jung JY, Park MY, Kim SK, Jung CK, Chung YG (2016). Differentiation of malignant from benign soft tissue tumours: use of additive qualitative and quantitative diffusion-weighted MR imaging to standard MR imaging at 3.0 T. Eur Radiol.

[18] Mastoraki A, Strigkos T, Tatakis FP, Christophi A, Smyrniotis V (2015). Recurrent low-grade Fibromyxoid sarcoma of the neck: report of a case and review of the literature. Indian Journal of Surgical Oncology.

[19] Oda Y, Takahira T, Kawaguchi K, Yamamoto H, Tamiya S, Matsuda S, Tanaka K, Iwamoto Y, Tsuneyoshi M (2004). Low-grade fibromyxoid sarcoma versus low-grade myxofibrosarcoma in the extremities and trunk. A comparison of clinicopathological and immunohistochemical features. Histopathology.

[20] Billings SD, Giblen G, Fanburgsmith JC (2005). Superficial low-grade fibromyxoid sarcoma (Evans tumor): a clinicopathologic analysis of 19 cases with a unique observation in the pediatric population. Am J Surg Pathol.

[21] Fisher C, Thway K (2014). Aggressive fibromatosis. Pathology.

[22] Diviti S, Gupta N, Hooda K, Sharma K, Lo L (2017). Morel-Lavallee lesions-review of pathophysiology, clinical findings, imaging findings and management. J Clin Diagn Res.

